# Oxidative Stress and Endoplasmic Reticulum Stress in Rare Respiratory Diseases

**DOI:** 10.3390/jcm10061268

**Published:** 2021-03-18

**Authors:** María Magallón, Sara Pastor, Ana Esther Carrión, Lucía Bañuls, Daniel Pellicer, Silvia Castillo, Sergio Bondía, María Mercedes Navarro-García, Cruz González, Francisco Dasí

**Affiliations:** 1 Research Group on Rare Respiratory Diseases (ERR), Department of Physiology, School of Medicine, University of Valencia, Avda. Blasco Ibáñez, 15, 46010 Valencia, Spain; mariamagallon94@gmail.com (M.M.); spastor@uic.edu (S.P.); carrionanaesther@gmail.com (A.E.C.); lucia.banyuls.soto@gmail.com (L.B.); dpellicerroig@gmail.com (D.P.); 2 Research Group on Rare Respiratory Diseases (ERR), Instituto de Investigación Sanitaria INCLIVA, Fundación Investigación Hospital Clínico Valencia, Avda. Menéndez y Pelayo, 4, 46010 Valencia, Spain; sccorullon@gmail.com (S.C.); sergibondia@gmail.com (S.B.); mer_navarro2002@yahoo.es (M.M.N.-G.); cruz.gonzalez@uv.es (C.G.); 3 Paediatrics Unit, Hospital Clínico Universitario de Valencia, Avda. Blasco Ibáñez, 17, 46010 Valencia, Spain; 4 Pneumology Unit, Hospital Clínico Universitario de Valencia, Avda. Blasco Ibáñez, 17, 46010 Valencia, Spain

**Keywords:** oxidative stress, endoplasmic reticulum stress, antioxidant therapies, rare respiratory diseases, Alpha-1 antitrypsin deficiency, idiopathic pulmonary fibrosis, cystic fibrosis, primary ciliary dyskinesia

## Abstract

Several studies have shown that some rare respiratory diseases, such as alpha-1 antitrypsin deficiency (AATD), idiopathic pulmonary fibrosis (IPF), cystic fibrosis (CF), and primary ciliary dyskinesia (PCD) present oxidative stress (OS) and endoplasmic reticulum (ER) stress. Their involvement in these pathologies and the use of antioxidants as therapeutic agents to minimize the effects of OS are discussed in this review.

## 1. Introduction

Oxidative stress (OS) is defined as an imbalance between pro-oxidant and anti-oxidant substances favouring the former [1]. In the physiological state, Reactive Oxygen Species (ROS) are necessary to neutralise pathogens that may attack the organism. One of their functions is to activate inflammatory intracellular signalling pathways, leading to the immune system activation [2]. However, when ROS appear in excess and accumulate within the cells, they create a highly oxidative state that can cause severe and irreparable damage to the tissues either directly or through altering signaling pathways [3]. Endoplasmic reticulum (ER) stress is the consequence of the accumulation of misfolded proteins in the ER lumen. Consequently, the ER activates the unfolded protein response (UPR), which leads to the elimination or repair of these proteins [4].

Considerable evidence has shown that oxidative and ER stress play an essential role in the pathophysiology of multiple disorders, including rare respiratory diseases, a group of hereditary disorders affecting the respiratory tract, characterized by low incidence and very heterogeneous symptoms [5]. 

In this review, we summarize the implications of ER and OS in the pathophysiology of four of the most common rare respiratory diseases: alpha-1 antitrypsin deficiency (AATD), idiopathic pulmonary fibrosis (IPF), cystic fibrosis (CF), and primary ciliary dyskinesia (PCD), for which there is no definitive treatment. In addition, since considerable evidence indicates that OS and ER stress could be potential therapeutic targets for these conditions, the current status of antioxidant therapies for treating these diseases will be discussed.

## 2. What Is Oxidative Stress?

Free radicals are molecules with an unpaired electron in their outer orbital; therefore, they are prone to react with other molecules to obtain the electron they need to reach their electrochemical stability [6]. Thus, free radicals can react with DNA, lipids, and proteins, leading to oxidation and, generally, to the loss of these biomolecules’ activity [7]. 

As a product of aerobic cell metabolism, two basic types of free radicals are generated in cells. Reactive oxygen species (ROS) include the hydroxyl radical (OH.), the superoxide anion (O_2_^−^), the hypochlorite ion (OCl^−^), and hydrogen peroxide (H_2_O_2_); as well as reactive nitrogen species (RNS) such as nitric oxide (NO) and peroxynitrite (ONOO^−^) [8]. The main sources of intracellular ROS and/or RNS generation are mitochondria, lysosomes, peroxisomes, xanthine oxidase, cytochrome P450, and the ER. Free radicals are also generated by exposure to external factors such as cigarette smoke, ionizing radiation, UV radiation, or environmental toxins [9,10].

Eukaryotic cells possess some mechanisms that diminish oxidative damage caused by ROS. The most straightforward defence mechanism involves small molecules such as reduced glutathione (GSH) and dietary components (i.e., vitamins, lipoic acid, and carotene), which oxidate themselves, thereby protecting the biomolecules. A more complex defence mechanism involving enzymes such as superoxide dismutase (SOD), catalase (CAT), glutathione peroxidase (GPx), and glutathione reductase (GR), has evolved to reduce ROS levels [7,11,12].

Under physiological conditions, a balance exists between production and degradation of ROS. When the balance between pro- and anti-oxidants shifts in favour of the former, a condition known as OS is produced, linked to the development of numerous diseases. Overproduction of ROS, induced by various exogenous and endogenous cellular sources, depletes antioxidant capacity and contributes to developing several disease-related processes. When the defence mechanisms cannot prevent ROS accumulation, the activation of specific signalling pathways causes changes in gene expression and protein synthesis. All these effects led to the hypothesis that increased free radicals and oxidative damage cause an increase in cell damage, which leads to the development of various pathological conditions, such as carcinogenesis [13], chronic inflammation [14], ageing [15], autoimmunity [16], cardiovascular diseases [17], neurodegenerative diseases [18], and respiratory diseases [3], among others.

However, OS cannot be only defined by a quantitative disbalance between reactive species and antioxidant defence mechanisms. OS is a more complex concept, besides the increment of ROS, other features such as their cellular, subcellular, or tissue location, chemical nature, the kinetics of formation and degradation, and time of exposure should be considered. Moreover, even in the absence of OS, basal levels of ROS exist in the cells. ROS act as regulatory and signalling molecules and are essential to proper cell function in a system known as REDOX regulation [19], participating in cell division, differentiation, and death. Consequently, OS also produces dysregulation of the redox signalling, and therefore, an alteration in cellular homeostasis [20,21].

Specific cell mechanisms remove oxidized lipids and oxidized proteins. However, oxidised DNA cannot be replaced and has to be repaired [22]. In response to the oxidative DNA damage, the cell reacts through several mechanisms, such as repairing these lesions; activation of control points of the cell cycle, which produces cell cycle arrest and prevents the transmission of damaged chromosomes; and apoptosis [23].

As a result of the various molecular oxidation processes, some products are produced and released to the extracellular media and can be used to measure the redox state. Three approaches are used to measure OS. The first approach involves determining oxidative damage to biomolecules, including lipids, proteins and nucleic acids. The most representative markers of lipid peroxidation include isoprostanes and malondialdehyde (MDA) [24], while carbonyl groups reflect the oxidative modification of proteins [25], and mutagenic and modified base 8-hydroxy-2′-deoxyguanosine (8-OHdG) reflecting DNA oxidation [26] products, which can be either nuclear or mitochondrial. A second approach involves the direct determination of ROS/RNS. Finally, a third approach involves measuring the enzymatic and non-enzymatic antioxidant systems (oxidized glutathione/reduced glutathione, dietary vitamins, and oligo-elements).

## 3. Clinical Relevance of Oxidative Stress 

**Figure 1:**
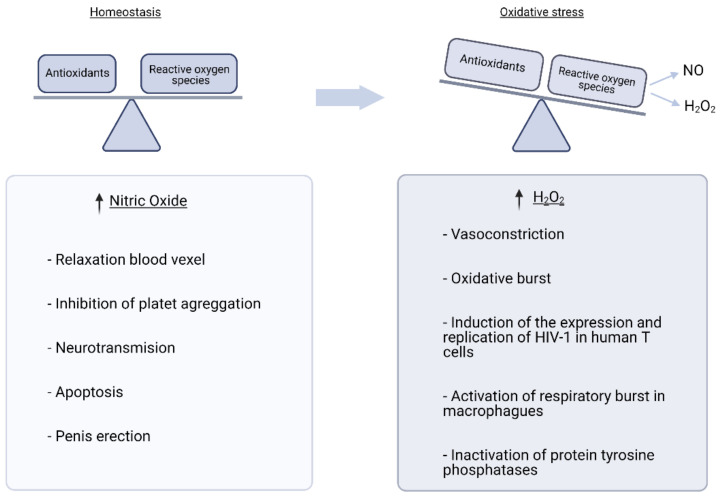
Clinical relevance of reactive oxygen species. Reactive oxygen species (ROS) have numerous functions involved in maintaining cellular homeostasis, such as those shown in the figure. However, when ROS levels increase, these functions are altered by dysregulation of signalling pathways.

Redox homeostasis involves a wide range of substances. As mentioned above, the balance of pro-oxidant and anti-oxidant substances is conditioned by many variables, which are complex to understand. Oxidative damage is the endpoint in which the cells’ biomolecules are oxidized and lose their functionality (Figure 1) [27]. 

Given the complexity of the oxidant-antioxidant pathways and their relationships, it is essential from a clinical perspective to understand the cause of the OS and its source because various free radicals act differently, causing different cell responses. It was initially thought that all free radicals could act as second messengers. However, further research showed that only H_2_O_2_ and other hydroperoxides fit the requirements to be considered as second messengers [28]. Among the RNS, NO is a well-known second messenger [29]. Other free radicals are not second messengers, although they trigger adaptive cell responses [28].

Second messengers are the intracellular component through which cells or organs interact with each other [30]. They should have a significant specificity with effectors from signalling pathways, and the reaction should be fast enough to not react with other molecules [28]. The hydroxyl radical does not fit the specificity requirement because it can oxidize any organic molecule [31], nor does the superoxide radical because the reduction of O_2_^−^ to H_2_O_2_ by SOD [32] occurs in the cell more rapidly than the interaction with the signalling pathways effectors [28]. When O_2_^−^ has been produced extracellularly (as in NADPH oxidases (NOX)), it can move into the cell and alter signalling pathways [33]. Even though O_2_^−^ cannot be considered a second messenger, it oxidizes GSH and other thiols, but it only produces a physiological effect when there is little SOD present, such as in phagosomes or endosomes [34]. GSH can scavenge O_2_^−^, but it generates thiol radicals, which further react to generate O_2_^−^ and subsequently H_2_O_2_ [34]. Therefore, O_2_^−^ might be considered as an H_2_O_2_ precursor [28,31].

H_2_O_2_ oxidizing potential can occur in two different ways. The first one is a one-electron reduction reaction, where a transition metal reduces H_2_O_2_. For instance, in the Fenton reaction, Fe^2+^ reduces the H_2_O_2,_ forming a hydroxyl radical plus Fe^3+^. As stated above, the hydroxyl radical is not a second messenger because it can react with any organic molecule, so it is not specific enough to be involved in controlled cell signalling [28]. Conversely, H_2_O_2_ can be reduced through another mechanism, the two-electron nucleophilic substitution reaction, where a thiol is converted to a thiolate anion, which reacts with the neighbouring cysteine residues of proteins such as peroxiredoxins. This reaction allows H_2_O_2_ or any peroxide to act as a second messenger [19]. 

Depending on the cell type targeted by H_2_O_2_, different cell responses can be achieved. It was demonstrated that exogenous addition of H_2_O_2_ in T cells activates the nuclear factor-κB (NF-κB) transcription factor [35], as does endogenous production [36]; a protein kinase C (PKC) is upstream of this signalling pathway [37]. In addition, hydroperoxides and H_2_O_2_ are linked with the respiratory burst in macrophages. At high concentrations, hydroperoxides inhibit the respiratory burst without killing the cells [38], but low concentrations stimulate it [39] by increasing intracellular Ca^2+^ levels [40] and activating the phosphatidylcholine specific to phospholipase C [41]. The oxidative burst activation in the extracellular-regulated kinase (ERK) pathway is also H_2_O_2_-dependent [42]. Likewise, the increase in the endogenous production of H_2_O_2_ causes a transitory inactivation of the protein tyrosine phosphatase 1B (PTP1B). PTP1B inhibits the activation of the ERK signalling pathway, dephosphorylating Raf1 [43]. This inactivation is reversible [44].

NO is a second messenger because it can pass across plasma membranes to exert its action in adjacent cells in a paracrine way. The NO mechanism of action consists of activation of guanylyl cyclase, which rapidly increases cyclic guanosine monophosphate (cGMP) concentration, leading to the phosphorylation of other proteins kinases that become involved in diverse cell functions such as relaxation of blood vessels, apoptosis, or penile erection [45].

OS parameters can be used as non-invasive diagnostic [46] or prognostic biomarkers because, as previously stated, in different diseases, free radicals have different effects. Although numerous studies have attempted to achieve this, limitations in the sample size or the fact that the study was a meta-analysis with a high heterogeneity among the studies have not allowed the establishment of a cut-off point for the use of OS parameters in rapid molecular risk stratification and outcome prediction. To overcome these limitations, extensive multicentric longitudinal studies with a larger sample size should be performed.

## 4. Endoplasmic Reticulum Stress

ER stress is the consequence of a mismatch between the load of unfolded and misfolded proteins in the ER and the cellular machinery’s capacity to cope with that load [4].

Protein folding occurs in the ER lumen, and its efficiency depends on several intrinsic and extrinsic factors, such as the ER environment, gene mutations, or altered posttranscriptional modifications. Every protein, before being secreted, must pass a quality control to check that it is correctly folded. Misfolded proteins are retained in the ER lumen to be correctly folded or degraded. Accumulation of unfolded/misfolded proteins leads to ER stress. This complex situation activates the UPR using three main approaches: increased ER capacity to fold and modify proteins, decreased global mRNA translation, and activated ER-associated degradation (ERAD) and autophagy. When UPR cannot solve the problem, it becomes chronic, and cell death is promoted by the activation of the pro-apoptotic signalling machinery.

**Figure 2:**
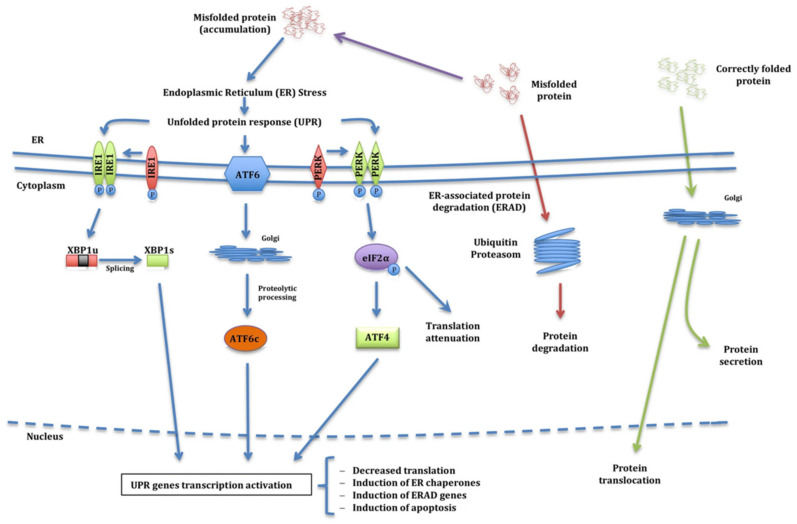
Signalling pathways of the Unfolded Protein Response (UPR). In a normal situation inside the endoplasmic reticulum, proteins are folded and taken to the places where they carry out their function (green arrows). When proteins are not folded correctly, they are stored in the lumen of the endoplasmic reticulum (ER), and the ER-associated protein degradation machinery (ERAD) is activated (red arrows). Occasionally, the ER shows dysfunctions that cause malformed proteins not to be degraded by ERAD and the proteins associate with each other creating aggregates, producing what is known as ER stress (blue arrows). When this occurs, the cell activates the UPR, which might mean that accumulated misfolded proteins can be detected by inositol-requiring enzyme 1 (IRE1), which activates transcription factor 6 (ATF6) and protein kinase R-like endoplasmic reticulum kinase (PERK) proteins.

ER stress activates three different signalling pathways of the UPR: inositol-requiring protein-1 (IRE1), activating transcription factor-6 (ATF6), or protein kinase R-like endoplasmic reticulum kinase (PERK) (Figure 2).

Consequently, IRE1 dimerises and autophosphorylates, activating its endoribonuclease activity, removing an intron of the transcription factor X-box-binding protein 1 (XBP1u) converting it into XBP1s, which activates transcription of UPR target genes. On the other hand, and as a consequence of the accumulation of these unfolded proteins, the ER can also activate ATF6, which splits and activates the transcription factor ATF6c, which travels to the nucleus and activates UPR genes. Finally, another option for UPR pathway activation is PERK activation. PERK dimerises and phosphorylates eukaryotic initiation factor 2α (eIF2α), which activates the transcription factor ATF4 that, like the previous ones, targets the nucleus to activate UPR gene transcription. Nevertheless, UPR genes can also decrease translation by preventing the accumulation of more misfolded proteins. They can also induce activation of ER chaperones to increase protein folding capacity, induce transcription of ERAD genes to increase degradation capacity, and activate apoptosis of ER-presenting cells if needed. Reproduced with permission from Torres-Durán et al. [47] under the terms of the Creative Commons Attribution 4.0 International License (http://creativecommons.org/licenses/by/4.0/ (accessed on 17 March 2021)). 

The ATF6 pathway is activated by unfolded/misfolded proteins sequestering the immunoglobulin heavy chain binding protein (BiP). BiP was previously attached to ATF6, and the sequestration of BiP triggers that signalling pathway [48]. IRE1 and PERK have related and interchangeable sensing domains when activating homodimerize, although the activation mechanism is not fully understood. Three different hypotheses have been proposed [4]: direct recognition, where the luminal domain directly binds the unfolded proteins [49]; indirect recognition, in which BiP is attached to IRE1 and PERK [50], and finally the hybrid recognition model, where both BiP dissociation and unfolded protein binding cause the activation of the signalling pathways [51].

### 4.1. ATF6

ATF6 is a regulatory protein that binds to ER stress-response elements (ERSE), a consensus sequence (CCAAT-N9-CCACG) that promotes UPR responsive genes. It has two homologous proteins: ATF6α (90 kDa) and ATF6β (110 kDa); both are synthesized in all cell types as ER transmembrane proteins [52]. In normal conditions, ATF6 is attached to the ER membrane-bound to BiP, but when the unfolded proteins accumulate, BiP dissociates from ATF6, and ATF6 goes to the Golgi complex, where it is cleaved by two proteases [48]. Serine protease site-1 (S1P) cleaves ATF6 in the luminal domain, whereas metalloprotease site-2 (S2P) cleaves the N-terminal portion within the phospholipid bilayer [53]. These reactions release the basic leucine zipper (bZIP) domain, which translocates to the nucleus to activate transcription. ATF6 binds to ATF/cAMP response element (CRE) and ERSE [54] to upregulate the expression of refolding genes [55].

### 4.2. IRE1

IRE1 was the first-identified component of the UPR, and it is an atypical type I protein kinase endoribonuclease, with a luminal dimerization, a cytosolic kinase, and an endonuclease domain [56,57,58]. When unfolded proteins are detected, IRE1 homodimerizes and trans-autophosphorylates to activate its RNAse domain. The IRE1p endoribonuclease substrate was first discovered in yeast. It is an mRNA that encodes bZIP, containing the transcription factor Hac1p [56]. The homologue in mammals is X-box binding protein (XBP1) [59,60], which is cleaved by IRE1 to remove an intron that causes frameshift. This splicing causes a change in the C-terminal region of XBP1, and only the spliced form of XBP1, XBP1(S), is a transcriptional factor involved in a variety of UPR target genes [61], both refolding and degrading genes [55].

### 4.3. PERK

PERK is a type I transmembrane protein with a kinase domain. During ER stress, PERK homodimerizes and trans-autophosphorylates (same way as IRE1). The C-terminal cytoplasmic domain of activated PERK directly phosphorylates the Ser51 of eukaryotic initiation factor 2 (eIF2α), attenuating global protein synthesis, thereby reducing the ER protein-folding burden. PERK activation occurs within minutes after developing ER stress [62]. Phosphorylated eIF2 α is required for selective translation of a subset of mRNAs. One important transcription factor activated by eIF2 α is transcription factor 4 (ATF4), which promotes the transcription of genes involved in amino acid biosynthesis, antioxidant responses, ER chaperones, growth arrest, DNA damage 34 (GADD34), and CAAT/enhancer-binding protein (C/REB) homologous protein (CHOP).

## 5. Endoplasmic Reticulum Stress and Oxidative Stress

**Figure 3:**
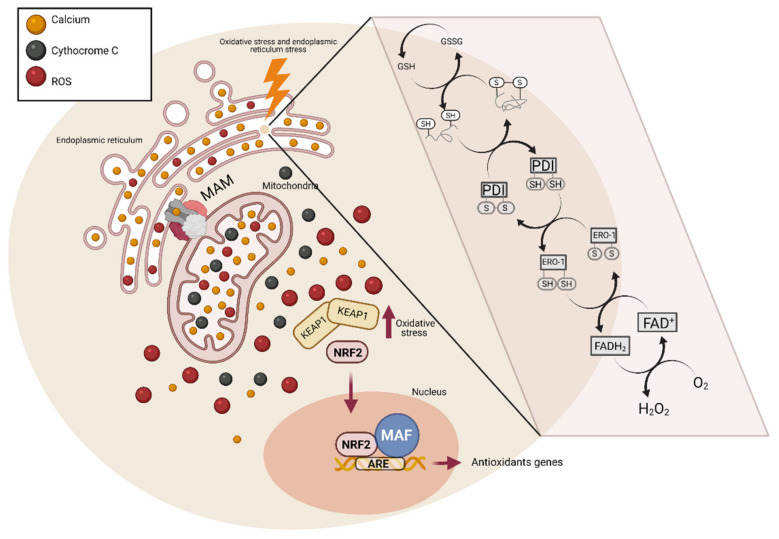
The connection between oxidative stress and endoplasmic reticulum stress. The presence of reactive species inside the endoplasmic reticulum favours the formation of the disulphide bonds in the protein folding. When this occurs, two electrons are released and accepted by protein disulphide isomerase (PDI), which loses its original conformation, accumulating inside the endoplasmic reticulum (ER) and triggering ER stress. PDI then releases two more electrons that are accepted by ER oxidoreductase (ERO1). Finally, the electrons are accepted by O_2_, leading to the production of H_2_O_2_. An increase in H_2_O_2_ causes Ca^2+^ levels in the ER to increase. The ER and mitochondria are linked by channels called MAM. When Ca^2+^ increases in the ER, it moves to the mitochondria. Elevated Ca^2+^ levels in the mitochondria stimulate mitochondrial metabolism, producing even more ROS. Ca^2+^ also increases the permeability of the mitochondrial membrane, allowing cytochrome C to be released and activate cellular apoptosis pathways. The increased ROS levels induce the release of *Nrf2* from Keap1, translocates to the nucleus where it binds to an accessory protein, Maf. The complex formed by *Nrf2* and Maf leads to the activation of antioxidant genes, interacting with an antioxidant response element (ARE).

Several studies have shown a link between ER and OS together (Table 1), but this relationship’s mechanism is still not completely understood. For clarity, we have separate it into three sections: (i) ER oxidative environment for disulfide-bond-forming; (ii) cross-talk between ER, mitochondria, and UPR, and (iii) activation of antioxidant genes (*Nrf2*) (Figure 3).

In physiological conditions, the ER environment is between 10 and 100 times more oxidative than the cytosolic compartment [63]. This oxidative environment of the ER favours protein folding, particularly forming disulfide bonds between two cysteine residues with the generation of H_2_O_2_. Disulfide bond formation is a reversible process achieved by a thiol-disulfide exchange reaction; this stabilizes the tertiary and quaternary protein structures [64]. It has been suggested that increased H_2_O_2_ levels oxidize and inactivate ER-resident proteins, such as protein disulfide isomerases (PDI), contributing to unfolded protein accumulation [65]. During disulfide bond formation, PDI accepts two electrons from the cysteine residues in polypeptide substrates, leading to the reduction of PDI and oxidation of the protein substrate. Then, PDI transfers the electrons to another acceptor, ER oxidoreductase 1 (ERO1), starting another cycle of disulfide bond formation. Then, ERO1 transfers the electrons to molecular oxygen (O_2_) to produce H_2_O_2_, the major ROS produced in the ER lumen [61,64]. It was calculated that 25% of the cellular ROS is the H_2_O_2_ produced in the ER by ERO1 folding activity [66].

The ER is the principal intracellular reservoir of calcium that controls Ca^2+^ homeostasis. However, more actors directly or indirectly play this role, such as mitochondria, pyruvate, isocitrate, and α-ketoglutarate dehydrogenases [67]. The ER and mitochondria are physically connected by mitochondrial-associated membranes (MAM), where membrane and luminal components can be exchanged [68]. The MAM composition depends on internal and external stimuli. The formation and destruction of mitochondrial associated membranes (MAMs) depend on changes in organelle dynamics [69]. Under ER stress conditions, CHOP expression increases in the ER’s cytosolic membrane, favouring the formation of a complex between CHOP and the mitochondrial translocase, translocase of outer mitochondrial membrane 22 (Tom22), either directly or through steroidogenic acute regulatory protein (StAR). The formation of this complex allows a stronger interaction between Tom22 and 3β-hydroxysteroid dehydrogenase type 2 (3βHSD2) increasing steroid metabolism [70,71]. Moreover, the inositol 1,4,5-trisphosphate receptor (IP3R), the voltage-dependent anion channel (VDAC), and the chaperon glucose-regulated protein 75 (GRP75) form a complex that provides the main Ca^2+^ transfer channel in MAMs [72]. Verfaille et al. suggested PERK as a novel component of MAMs in the ER surface. The perturbations of the ER/mitochondria contact sites reduce the propagation of ROS signals to the surrounding mitochondrion, attenuating the onset of apoptosis provoked by ROS-based ER stress [73]. This structure has lipids and proteins that suggest a two-way supply of fundamental metabolites and messengers that control mitochondrial function, thereby controlling the bioenergetics rate [69]. 

Bravo et al. showed that in early ER stress stages, MAMs increase, so the Ca^2+^ transfer increases from ER to mitochondria, thus enhancing mitochondrial respiration, reductive power, and ATP production [74]. Moreover, mitochondrial Ca^2+^ uptake stimulates the activity of some Krebs cycle enzymes both directly (isocitrate and α-ketoglutarate dehydrogenases) and indirectly (pyruvate dehydrogenase) [75], which, in time, increases O_2_ consumption, resulting in an ROS increase [61]. Ca^2+^ opens the permeability transition pore so that cytochrome c can be released, blocking the respiratory chain complex III, which increases O_2_^−^ production [76]. Wang et al. showed that chronic ER stress oppositely modulates cellular metabolism, decreasing the mitochondrial metabolism, lowering the mitochondrial membrane potential and the mitochondrial mass [77].

The nuclear factor erythroid-derive-2 (*Nrf2*) protein is a bZIP transcription factor characterized by its conserved structural domain, referred to as the cap‘n’collar (CNC) domain. These CNC transcription factors function as heterodimers, binding to accessory proteins such as Mafs to activate gene expression [78]. *Nrf2* binds an antioxidant response element (ARE), a cis-element in the promoters of many anti-oxidative genes that is crucial to their inducible activation [79]. Under non-stressed conditions, *Nrf2* persists at low levels in the cytoplasm, where it is bound to its inhibitor, Keap1 [80]. Keap1 serves to anchor *Nrf2* in the cytoplasm and signal its ubiquitination and subsequent proteasomal degradation, resulting in low baseline expression of the *Nrf2*-dependent genes. However, the disulfide bonds in Keap1 are susceptible to OS, and exposure to a wide variety of electrophiles/oxidants triggers a conformational change in Keap1, caused by the modification of thiol residues, releasing *Nrf2*. Other post-translational modifications also facilitate this dissociation, including phosphorylation of *Nrf2* and S-nitrosylation of Keap1.

Upon dissociation from Keap1, *Nrf2* translocates to the nucleus, heterodimerizes with Maf proteins, binds ARE, and activates the coordinate expression of hundreds of genes. The net result is an adaptive cytoprotective response that detoxifies stressors.

PERK-dependent phosphorylation triggers the dissociation of *Nrf2*/Keap1 complexes and inhibits the reassociation of *Nrf2*/Keap1 complexes in vitro. Activation of PERK via agents that trigger the UPR is necessary and sufficient for the dissociation of cytoplasmic *Nrf2*/Keap1 and the subsequent *Nrf2* nuclear import [81]. *Nrf2* activation contributes to the maintenance of GSH levels, which functions as a buffer of ROS accumulation during the UPR. The nocive effects of *Nrf2* or PERK deficiencies could be attenuated by the restoration of cellular GSH levels or *Nrf2* activity. The inhibition of ROS production attenuates apoptotic induction following ER stress. These data suggest that perturbations in cellular redox status sensitize cells to the harmful effects of ER stress [82].

**Table 1:** Summary of some studies where endoplasmic reticulum (ER) stress and oxidative stress (OS) are present simultaneously.

Cell Type	Stimulus	ER Stress Measurement	OS Measurement	ER–OS Branch	Observation	Ref
**PERK-/- and PERK+/+ MEFs**	Phox-ER stress	CHOP and Chaperones ↑	DiOC6 ↓ (Ψm) NAO ↑ (oxidized cardiolipin)	Cross-talk ER-mitochondria	PERK is a component of MAMs.	[73]
**Bax-/- haemopoietic cells**	Tunicamycin	CHOP ↑ BiP/GRP78 ↑	Mitotracker Red ↓ (Ψm) → ↑mitochondrial O_2_^−^	Cross-talk ER-mitochondria	Mitochondrial mass, O2 consumption & ATP production ↓.	[77]
**S. cerevisiae Hip deficient cells (erv29∆)**	CPY (a misfolded mutant form of protein carboxypeptidase)	IRE1 ↑	DHR-123 ↑ (General ROS)	Antioxidant genes	GSH suppress ROS and cell death but not ER stress.	[83]
**CHO cells**	Misfolded factor VIII expression	BiP ↑ eIF2α-P ↑ CHOP ↑	DCF ↑ (peroxides), DHE ↑ (superoxide), MDA ↑, GSH ↓, GSSG ↑ HODE ↓ (hydroxioctadecaidienoic acid) Prot. Carbonyls ↓	Antioxidant genes	BHA (butylated hydroxyanisole) antioxidant ↓apoptosis, ↓intracellular accumulation of misfolded proteins and ↑secretion of properly folded proteins.	[84]
**PERK-/- and ATF4-/- fibroblast and** **C. elegans PERK -/-**	Tunicamycin	Several genes	DCF ↑ (peroxides)	ER oxidative environment for disulfide bond forming	ATF4-/- cells are impaired in expressing genes involved in aa import, GSH synthesis, and OS resistance. PERK-/- cells accumulate endogenous peroxides during ER stress, whereas interference with the ER oxidase ERO1 abrogates such accumulation. eIF2α phosphorylation protects cells against metabolic consequences of ER oxidation by promoting the linked processed of sufficiency and resistance to OS.	[85]
**Left ventricle cells form five-month-old Lee-Sung (Met-S) and Lanyu (MHO) obese minipig**	High-fat diet	CHOP ↑ (Met S & MHO) PERK ↑ (Met S & MHO) IRE1α = (Met S & MHO) ATF6 (↑ Met S & = MHO)	TBARS (Thiobarbituric acid reactive substances) (↑ Met S & ↑ MHO)			[86]
**Primary murine brain endothelial cells from 2 month-old BL/6 mice**	T-BHP (Tert-butyl hydroperoxide)	XBP1-S ↓ CHOP ↑	DCF ↑ (peroxides) MDA ↑ 4-HNE ↑ CAT = SOD = GPx = RH ↑ (Ψm)	Cross-talk ER-mitochondria	Down-regulation of Homer1 protects against t-BHP-induced endothelial injury. Down-regulation of Homer1 reduces t-BHP-induced OS. Down-regulation of Homer1 preserves Ca^2+^ homeostasis in mBECs. Down-regulation of Homer1 attenuates t-BHP-induced ER stress.	[87]
**Human PBMCs cognitive impairment.** **PBMCs and brain cortex cells from a transgenic mouse with Alzheimer’s disease**	Thapsigargin	GRP78 ↑ XBP1 ↑	DCF ↑ (peroxides) Nrf2 ↑ GCLc	Antioxidant genes		[88]
**MIA PaCa-2 human pancreatic cells**	Piperlongumine	ATF4 ↑ IRE1α ↑ XBP1 ↑	OSGIN1 ↑ ABCB10 ↓			[89]
**Hepatopancreas from *Litopenaeus vannamei***	Ammonia nitrogen	BiP ↑ eIF2α = ATF4 ↑ IRE1 = XBP1-S ↑	SOD ↓ MDA ↑			[90]

↑: Indicates increased expression or production; ↓: Indicates decreased expression or production; = Expression or production are not modified

Malhotra et al. showed both in vivo (mice) and in vitro (CHO-H9 cells) that antioxidant treatment reduced ER stress and the associated OS, and protein secretion was improved [84]. Therefore, even though it is unknown how the misfolded proteins accumulated in the ER produce ROS, these authors demonstrated that accumulated unfolded proteins are sufficient to produce ROS and that both unfolded proteins and ROS are required to activate UPR [84].

## 6. Endoplasmic Reticulum and Oxidative Stress in Rare Respiratory Diseases

### 6.1. Serpinopathies, Endoplasmic Reticulum, and Oxidative Stress

Serpins are a protein superfamily of around 350–500 amino acids distributed in the metazoan, plantae, and certain viruses [91]. They have a similar structure with a high homology in sequence and alike structures. Their primary function is to inhibit proteases, but studies using model organisms have shown that serpins also control proteolysis in molecular pathways associated with cell survival, development, and host defence. Non-inhibitory serpins are described as essential elements with diverse biological systems serving as chaperones, hormone transporters, or anti-angiogenic factors [92]. Serpins are vulnerable to mutations that lead to protein misfolding and polymerization of mutant proteins frequently occurs, reducing the number of active inhibitors and leading to the accumulation of polymers, causing cell death and organ failure [93]. These diseases are called serpinopathies.

The most studied serpinopathy is AATD (ORPHA:60), a rare genetic disease with a prevalence of 1–5/10,000. In this case, the serine protease inhibitor is alpha-1 antitrypsin (AAT), which is mainly synthesized and secreted by hepatocytes. AAT’s main function is to protect lung tissues from neutrophil elastase [94]. Z-AAT is the deficient variant with the most clinical relevance leading to the formation of polymers that accumulate in the hepatocytes, producing severe liver disease in some patients. The lack of circulating AAT predisposes to emphysema.

There are some AATD studies related to ER stress, and it is unclear if Z polymers activate the UPR. Some studies showed that Z polymers do not cause UPR, nor in CHO-K1 expressing human Z-AAT [95], nor in HeLa cells [96] or rat liver [96,97], but they do in human peripheral blood monocytes [98] and HepG2 cells [99], which could be explained by the UPR needing secondary stress to activate the UPR. Lawless et al. reported that in CHO cells, the expression of the Z-AAT polymer alone does not lead to UPR, but when they added thapsigargin (an ER stressor) or heat stress, UPR was triggered [100]. Ordónez et al. supported the theory of the second stressor to activate the UPR. They observed that polymer-forming mutants of AAT (Z-AAT) only activate the ER overload response (EOR), whereas truncated AAT mutants only activate the UPR. These two pathways usually occur together. Their data revealed that polymers of AAT that accumulate in a spheric manner produce a loss of the normal tubule ER network, forming a vesiculated ER, which leads to impairment of luminal protein mobility [87]. ER vesiculation is associated with other cellular stresses, including mechanical injury and elevated cytosolic calcium concentration [101,102]. The truncated polymers cause classical ER stress (UPR) and are efficiently degraded by the proteasome, showing a different ultrastructural change characterized by gross expansion of ER cisternae. Z-AAT activate chaperones. The observation of enhanced sensitivity to ER stress following Z-AAT expression correlates with marked changes in the ER’s biophysical features. In cells experiencing ER overload, misfolded proteins cannot diffuse freely, decreasing their accessibility to the quality control required for folding and transportation. Conversely, in cells with reticular and highly interconnected ER, chaperones can diffuse to misfolded proteins’ sites. Therefore, a model is proposed in which decreased mobility or availability of ER chaperones due to changes in the diffusive features or/and obstruction caused by protein overload sensitizes the cell to subsequent activation of the UPR [96].

A study conducted in mice carrying the human mutant *Z-AAT* gene showed that protein aggregation does not trigger the elevation of the major stress proteins in UPR (calnexin, Gpr78, Gpr94, and PDI). The most abundant disulfide isomerase and chaperone in the ER, PDI, was found attached to the Z-AAT protein. Protein disulfide reductase (PDR) activity is predominantly performed in the ER by PDI, which is decreased in these transgenic mice, probably because of PDI sequestration in PiZ aggregates. PiZ mice were found to have more reduced ER, with a more considerable amount of reduced protein thiol groups, GSH, and GSH/GSSH ratio. The redox status in the cytoplasmic fraction was a little more oxidized, with the same amount of protein thiol groups and GSH but a slightly lower GSH/GSSG [97], which is consistent with our study, where we found that children with AATD have systemic OS, in part, through a decrease in GSH [103]. Thus, the shift in ER redox potential toward a reduced state promotes PDI acting as a chaperone rather than a disulfide isomerase [104]. The reduction of PDI disulfides and PDI’s decreased availability could explain the PDR deficiency of PiZ transgenic mice [97], which can be an adaptation of the ER, as found in other long-term stress models, such as diabetes [105]. Altogether, these data reveal a rescuing mechanism activated in long-term, nonlethal stress, during which a less productive, but more protective, steady-state of the ER is maintained, in which a more reducing environment protects the ER from OS and apoptosis and regulates PDI to act as a chaperone rather than an oxidoreductase [88]. To conclude, the study suggested a model for chronic ER stress, where different protective pathways are activated in contrast to short-term ER stress. The reduced ER environment, the change in PDI function, decreases in PDR activity, and the differences in chaperone complexes in the ER and chaperone and antioxidant enzyme induction in the cytoplasm, suggest a long-term adaptive response, which sacrifices efficient protein folding for long-term survival [97].

Some studies have linked OS and AATD. The first, performed in the PiZ mouse liver model, showed that Z transgenic mice experience oxidative was damage by increasing protein carbonylation, MDA, and 8-OHdG levels. This study also found that ageing liver tissue from older PiZ mice had elevated ROS and generally lower antioxidant enzyme levels than younger mice [106]. Another study showed that healthy children with AATD experienced increased oxidative damage caused by decreased GSH levels, decreased GSH/GSSG ratio and diminished CAT activity. Oxidative damage in lipids (MDA), DNA (8-OHdG), and carbonyl proteins was also observed [103]. Along this line, a report showed two PiZZ patients with severe emphysema and extremely high urine levels of 8-OHdG. The patient with the highest 8-OHdG also had a mutation in glutathione S-transferase pi 1(GSTP1) [107], an enzyme that plays an important role in detoxification by catalyzing the conjugation of many hydrophobic and electrophilic compounds with GSH [108]. A recent study showed that AATD patients with an intermediate and high risk of developing lung and/or liver disease were observed to have significantly shorter telomeres and increased oxidative damage than control individuals [109], indicating an association between telomere length and OS markers in AATD patients. 

AAT is a protein that acts as an antioxidant because it has nine methionine residues, which may protect proteins from oxidative damage [110]. These methionines can be oxidized, but mainly two (351 and 358) are prone to it. The oxidation of these two methionines results in the loss of antielastase activity [111]. Another study showed that exogenous AAT increases the antioxidant defence (SOD and GPx) and prevents preeclampsia development [112]. Then, deficient patients have less serum AAT and increased OS, which oxidizes the AAT and inactivates it, so the antielastase activity is even lower, increasing lung disease risk. Altogether, these findings suggest that OS is associated with AATD-related lung disease [103,107].

### 6.2. Interstitial Lung Diseases, Endoplasmic Reticulum, and Oxidative Stress

IPF is an interstitial lung disease (ILDs), a heterogeneous group of lung diseases characterized by inflammation and fibrosis. ILDs can be produced by exposure to environmental and pharmacological agents or sarcoidosis. Some patients have no identifiable cause, and the disease is classified as idiopathic interstitial pneumonia (IIPs). IPF is the most common form of ILD and one of the most aggressive forms of IIP [113].

The global IPF incidence ranges from 0.2 to 9.4 per 100,000 per year. The prevalence was estimated to be higher in men than in women (ORPHA:2032). IPF is a chronic disorder characterized by progressive fibrosis that leads to a severe decline in lung function, progressive respiratory failure, and high mortality [113]. The aetiology of IPF remains unknown; however, some pathogenic factors have been proposed: aberrant wound healing, profibrotic proteins (i.e., TGFβ), OS, and inflammation [114]. 

Recent studies have suggested that ER stress could also be involved in the pathogenesis of IPF. Various ER stress markers (i.e., ATF4, ATF6, CHOP BiP, EDEM, and XBP1) were found to be increased in alveolar epithelial cells (AECs) from IPF patients [115,116]. Fibroblasts in lung tissue from IPF patients show upregulated expression of BiP [117]. Alveolar macrophages from mice with asbestos-induced lung fibrosis and bronchoalveolar macrophages from asbestosis patients also showed increased BiP expression [118]. M2 macrophages from IPF patients were reported to express CHOP [119]. 

Based on the available data, ER stress could modulate several key components of lung fibrosis such as AEC apoptosis, myofibroblast differentiation, epithelial-mesenchymal transition (EMT), and M2 macrophage polarization [120]. Kamp et al. provided evidence that asbestos-induced ER stress can induce AEC apoptosis through IRE1 expression and ER Ca^2+^ release [121]. There is evidence of the influence of ER stress in cell differentiation. Baek et al. suggested that ER stress through UPR could induce differentiation of fibroblasts to myofibroblasts [117]. Other studies have reported that ER stress induces EMT in epithelial cells [122,123]. In a recent report using macrophages, Yao et al. showed that the ER stress might be able to induce M2 (pro-fibrotic phenotype) polarization through Jun N-terminal kinase (JNK) or CHOP in IPF [119].

Several studies have provided evidence of augmented OS in biological fluids and lung tissue from IPF patients. Oxidized proteins have been identified in the bronchoalveolar lavages (BAL) of IPF patients. Oxidation may lead to dysfunctional proteins, suggesting a pathological role of OS in IPF [124,125]. A remarkable increase in serum isoprostane levels was observed in IPF patients [126]. These findings suggest that increased OS and could be negatively correlated with the disease’s severity [127].

Additional studies showed higher ROS levels in IPF patients compared to healthy controls. In exhaled breath condensates (EBCs) from IPF patients, higher levels of H_2_O_2_ were determined [128]; pulmonary inflammatory cells were obtained from epithelial lining fluid (ELF) of IPF patients showed increased levels of ROS [129]. NOX, a family of pro-oxidant enzymes, was found to be upregulated in the lungs of IPF patients, and several studies have reported an increase in mitochondrial ROS generation [130,131].

The role of nitrosative stress in IPF has also been studied; NO seems to induce TGFβ- and ECM-degrading enzymes in fibroblasts in animal models of lung fibrosis [114,132]. Upregulated expression of inducible NO synthase (iNOS) was demonstrated in IPF epithelial cells, macrophages, and fibroblasts, which can provoke abnormal nitrosative stress, contributing to fibrogenesis [133].

The antioxidant defence is also altered in IPF patients. GSH is decreased in the alveolar ELF of the lower respiratory tract of IPF patients [134]. Several antioxidant enzymes, including SOD, are lower in fibroblast foci from IPF patients [135].

Increasing the expression of *Nrf2* in fibrotic lungs was not able to counteract the OS [136]. It has also been suggested that polymorphisms in *Nrf2* may participate in IPF susceptibility [137].

These alterations in redox signalling can affect the development of disease through different processes. ROS-induced DNA damage can lead to apoptosis of airway epithelial cells; ROS can increase the production of cytokines and TGFβ, which favours chronic inflammation, leading to progressive fibrosis [138].

### 6.3. Cystic Fibrosis, Endoplasmic Reticulum, and Oxidative Stress

CF is a rare autosomal recessive disease (ORPHA:586). The incidence of CF is currently 1:3500 [139]. It is a monogenic disease affecting the *CFTR* gene, located on chromosome 7 [140]. This gene codes for a type of ATP-binding cassette (ABC) transporter, whose function is to transport chloride and sodium ions and other anions such as GSH or bicarbonate (H_3_CO_3_). Located mainly in the apical membranes of epithelial cells in many tissues [141], CFTR can be affected by numerous types of mutations; more than 2000 variants of the *CFTR* gene are currently described [142]. Malfunctioning CFTR often affects ion conductance efficiency through the membrane pores [143], which changes the characteristics and composition of cellular secretions by changing the composition of the extracellular milieu. This accumulation of events causes organs to become gradually obstructed, eventually leading to fibrosis [140].

One of the main causes of CF is a mutation in the *CFTR* gene. The most common mutation is the loss of a phenylalanine at position 508, ΔF508 [144]. This sequence defect renders the protein unable to properly perform its transporter function, so it should be tagged by Hsc70-CHIP ubiquitin ligase and taken to the proteasome for degradation [145]. However, numerous studies have shown that the ER can fail in this process and produce an unwanted accumulation of CFTR protein, leading the cell to experience ER stress [144,146]. As discussed above, an accumulation of misshaped proteins leads to the activation of the UPR, which consists of three different but complementary signalling pathways: IRE1, ATF6, and PERK.

Kerbiriou et al. studied the relationship of CF pathogenesis in the activation of UPR pathways [147]. These researchers analyzed the presence of markers, showing that UPR signalling pathways were activated and studied ATF6, a bZIP transcription factor synthesized in the reticulum membrane that, during UPR activation, binds to the Golgi apparatus, converting itself into an active form and migrates to the nucleus, where it acts as a transcription factor generating a stress response [148]. Another marker they studied was Grp78, a glucose-regulated protein. Grp78 binds to the hydrophobic part of unfolded proteins and is related to the activation of ATF6, IRE1, and PERK, which makes it an exciting marker for studying UPR activation [149]. Finally, they showed that, as proposed, ATF6 and Grp78 levels were elevated in cells with mutated CFTR [147]. This could confirm the existence of ER stress in CF.

In another study, Tang et al. [150] aimed to find a relationship between the worsening of inflammation in CF patients and the activation of UPR pathways to find a therapeutic pathway involving ER stress in CF. They proposed that UPR signalling cascades lead to stimulation of the IκB kinase (IKK) through the interaction of tumour necrosis factor (TNFα) and IRE1α, leading to the activation of NF-κB. Nevertheless, they were unable to find such a relationship.

A CTFR abnormality in bronchial epithelial and ciliated cells leads to alteration of the pulmonary extracellular medium due to poor conduction of Na^+^, Cl^−^ ions, or GSH, resulting in excessive O_2_ consumption [151], causing hypoxia, among other effects [152] making the airways of CF patients a niche for bacterial infections [140]. This situation results in the activation of inflammatory MAPK pathways [140], leading to stimulation of NF-kB [153], which targets the nucleus and activates the cytokines IL-8 and IL-6 and TNFα [154]. These biomolecules attract the polymorphonuclear (PMN) cells of the immune system, the neutrophils, to the airway surface liquid (ASL). The primary function of neutrophils is to kill bacteria by phagocytosis. For the destruction of ingested material, neutrophils release ROS, such as O_2_^−^, H_2_O_2_, or free OH. resulting from NADPH’s oxidation [155]. Although neutrophil activation is a defence mechanism of the body, if ROS are not neutralized or controlled, they can cause irreparable damage in CF patients. Therefore, neutrophils are a significant ROS source in the ASL of children with CF [156,157].

Bronchial ciliated and type II AECs are the second source of oxidants in CF lungs through DUOX1 and DUOX2, two isoforms of the NOX family found in epithelial cells [158].

One of the main mechanisms of ROS neutralization is GSH. In 1993, Roum et al. demonstrated that GSH levels in CF patients are below those of healthy controls [159]. Years later, in 1998, Lindsell et al. studied the relationship of the CFTR channel to these decreased values, then demonstrated that CFTR, along with its chloride and sodium transport function, is also actively involved in the transfer of GSH from the intracellular to the extracellular environment [160] indicating that an alteration in the CFTR gene could lead to a decrease in GSH levels in the ASL, producing an oxidative imbalance. This hypothesis is now accepted and is proposed as one of the main reasons for OS in CF.

Other factors contributing to this OS, such as NO and H_2_O_2_, are currently being studied. It was shown that NO is decreased in CF patients’ bronchial airways, allowing an abnormal interaction of its mediator species with the surrounding environment, which would produce harmful effects [161]. Asymmetric dimethylarginine, an endogenous NOS inhibitor, is increased in CF airways [162], contributing to the reduced levels of NO in CF airways. Conversely, increased H_2_O_2_ levels have been found in different cultured epithelial cell models of CF, which are related to the elevated IL-6 and IL-8 production in CF epithelia [163]. These augmented H_2_O_2_ levels are associated with a lack of expression of regulatory agents due to the poor interaction between CFTR and *Nrf2*, a factor responsible for activating H_2_O_2_ regulatory mechanisms [163]. According to this finding, differential expression of antioxidant proteins was reported in cultured CF models compared with normal controls, such as TRX-1, GSTP1, peroxiredoxin (PRDX) 6, TRX-dependent peroxide reductase (PRDX-1), and CAT [163].

Biomarkers of oxidative damage have been reported in lipids and proteins from CF patients. Variations in lipid peroxidation have been found: MDA was demonstrated to be increased in plasma or serum from patients with CF [164] and 8-isoprostane was also found elevated in CF plasma [165], buccal mucosal cells [166], and breath condensate [167]. Oxysterols, a biomarker of cholesterol oxidation, are also increased in CF plasma [168]. Moreover, oxidative damage in proteins was observed in the airways of children with CF [157].

### 6.4. Primary Ciliary Dyskinesia and Oxidative Stress

PCD is a rare disease, with an estimated incidence of 1:20,000 live births (ORPHA:244). PCD is a genetically heterogeneous disorder. Disease-causing mutations in at least 40 genes have already been reported [169]. It is characterized by structural and/or functional alteration in motile cilia, which causes a deficit in the mucociliary clearance of the respiratory secretions [170] leading to chronic respiratory infections and chronic inflammation of the airways. An inefficient inflammatory process increases the number of neutrophils, increasing ROS and RNS, as explained above [171].

OS has rarely been studied in PCD. Altered RNS has been reported in PCD. Nasal NO (nNO) measurement is recommended as the initial test in patients with clinical suspicion of PCD since diminished nNO values are characteristic of patients with PCD [172]. However, despite its good sensitivity, there are cases of PCD with normal values of nNO, so even if the nNO is normal—if the symptoms are consistent—the disease should not be ruled out [173,174]. 

Patients with PCD suffer from oxidative damage, and increased levels of the OS marker, 8-isoprostane, has been observed in the exhaled breath condensate (EBC) of children with PCD compared to healthy controls [175].

A study of nasal epithelium cells from patients with PCD showed an alteration in the OS state compared to cells from healthy volunteers. Patients with PCD have lower apoptosis levels, NO, ONOO^−^, O_2_^−^, H_2_O_2_, and mitochondrial O_2_^−^ in nasal epithelium cells compared to healthy individuals [176]. Nevertheless, no significant differences were observed in the oxidative damage in lipids and proteins [176].

More studies are needed to determine the role that OS plays in the pathophysiology of the disease.

## 7. Antioxidants Therapies in Rare Respiratory Diseases

Due to the extensive evidence of oxidant/antioxidant imbalance and the role of OS in the pathogenesis of disease, several research groups have proposed antioxidants as promising therapeutic agents in AATD [177], IPF [178], and FQ [179].

### 7.1. Alpha-1 Antitrypsin Deficiency and Antioxidant Therapy

The only currently available specific treatment for AATD that currently exists is augmentation therapy, which consists of the intravenous administration of AAT purified from the plasma of healthy human donors [47]. This treatment has proved its clinical efficacy by delaying emphysema progression, protecting the lungs from excessive neutrophil elastase [47]. However, there had been controversy regarding the use of augmentation therapy and its efficacy [180] until the appearance of randomized, placebo-controlled trials, such as the RAPID trial [181]. Augmentation therapy requires regular intravenous infusion of AAT, which depends on the protein’s availability from donors. Therefore, to overcome these problems, other strategies are currently being investigated. 

More than 5% of patients diagnosed with chronic obstructive disease (COPD) are also diagnosed with AATD, and more than 40% of AATD patients develop COPD [47]. OS is one of the pathogenic mechanisms in COPD, as well as in AATD. OS enhances chronic inflammation and favours the appearance of emphysema. These findings have provided the possibility to study antioxidant therapies. Various studies have been conducted using GSH generating antioxidants, which have been observed to reduce exacerbations in COPD patients, although it has not been confirmed whether the benefit is due to their antioxidant or mucolytic properties. Various research groups are currently looking for more effective antioxidants; along this line, studies have been conducted using different types of antioxidants such as SOD mimetics, NOX inhibitors, mitochondria-directed antioxidants, or *Nrf2* activators [182]. The last one shows promise as a therapeutic target for COPD patients since it was observed that it is not increased in response to ROS as it does in normal cells. However, an antioxidant has yet to be found that demonstrates a clinical benefit for patients, so further studies evaluating the OS in lung tissue are required to identify more effective antioxidant therapies for COPD and, consequently, for AATD patients.

There is no specific treatment for AATD-related liver disease, other than liver transplantation, which has high associated risks [183]. Several studies have suggested that antioxidant therapy may modulate the secretion of AAT polymers into the bloodstream from hepatocytes. Ronzoni et al. suggested that the redox state in the ER could contribute to the retention of AAT, since the formation of disulfide bonds favours the accumulation of Z-AAT and other variants inside hepatocytes, being, therefore, OS, an AATD-modifying factor and a possible therapeutic target [184]. Studies reported that tobacco smoke induces the oxidation and polymerization of Z-AAT, which would explain emphysema’s premature appearance in smoking ZZ individuals. Using the antioxidant N-acetyl cysteine, the same authors managed to avoid the oxidation induced by tobacco extract in vitro and by the polymerization of Z-AAT [185].

### 7.2. Idiopathic Pulmonary Fibrosis and Antioxidant Therapy

ROS scavengers and drugs targeting redox imbalances might be promising strategies in treating IPF, among other targets such as senescence or the immune response. N-acetylcysteine (NAC) is a precursor to GSH and a free radical scavenger widely tested in the treatment of IPF. Various studies in animal models have proven the efficacy of this exogenous scavenger in mitigating bleomycin-induced fibrosis [186,187,188]. However, NAC did not show clear evidence of benefit in IPF patients [189]. Interestingly, specific patient subpopulations with the TOLLIP TT genotype show different responses, suggesting the need for personalized medicine in IPF [190]. 

Another therapeutic target studied for IPF is SOD, which is decreased in patients. A study on mice showed decreased bleomycin-induced fibrosis after intravenous administration of lecithinized SOD, a more biologically stable form of SOD [191]. However, in humans, a randomized controlled trial using lecithinized SOD in IPF patients showed no lung function improvement [192]. 

Novel targets for IPF have been investigated, targeting the redox imbalance. Hecker et al. suggested that GKT137831, a dual inhibitor of both NOX1 and NOX4, could be a promising therapeutic strategy in age-associated fibrotic disorders, able to reverse bleomycin-induced fibrosis in mice [193]. The anti-fibrotic role of metformin was also examined in a bleomycin-induced lung fibrosis model, being found to inhibit TGFβ1-induced NOX4 expression [194]. Azithromycin was found to enhance the proteasome degradation of NOX4 [195], and in another study in mice models, this compound was able to reduce bleomycin-induced fibrosis [196]. A single-centre, retrospective, observational study, carried out in IPF patients with acute exacerbation treat with azithromycin, indicated a possible improvement in mortality [197].

Activators of *Nrf2* might be promising as an IPF treatment as they are supported by several studies in animal models. Rapamycin seems to protect against paraquat-induced pulmonary fibrosis [198]. The ability of several substances, such as sulforaphane, pirfenidone, salidroside, and salvianolic acid B, to attenuate bleomycin-induced pulmonary fibrosis has been examined [199,200,201,202]. However, human trials are needed to evaluate the utility of *Nrf2* activators in IPF.

Another possible approach to IPF treatment is targeting ER stress. An example could be phenylbutyric acid (PBA), a chemical chaperone, which was found to inhibit EMT in the lungs, reduce the expression of pulmonary TGFβ1, and attenuate bleomycin-induced pulmonary fibrosis [203].

### 7.3. Cystic Fibrosis and Antioxidant Therapy

Antioxidants proposed as possible therapies include vitamin E, β-carotene, vitamin C, selenium supplements, and GSH or NAC [140,179].

GSH, both inhaled and orally, has been examined in numerous studies. Griese et al. showed that inhalation of GSH does not reduce inflammation nor OS [204]. Regarding the use of oral GSH, some controversy exists since some studies showed a reduction in OS [179], while others suggested the incompatibility that may exist between this treatment and those patients who have absorption difficulties, which is quite common in CF patients, opening up the possibility for further research with targeted therapies [140,205].

However, some of these studies suggested that it is difficult to obtain accurate results due to the effects of the treatment to which the patient is subjected, so repeating GSH and NAC treatments is proposed in children for a prolonged time [179].

Another common strategy in antioxidant research is the administration of NAC. Conrad et al. showed that NAC oral administration could, if not improve lung capacity, at least slow the process [206].

The use of vitamin E, vitamin C, or β-carotene, has also been extensively studied. These biomolecules have a great capacity as radical-scavenging antioxidants and could be used as neutralizers of free radicals [140,179,207]. However, no studies have analyzed their effects on redox imbalance in FC, leaving an open door for research with these substances.

Although still in the study phase, for selenium supplements, it has been observed that, together with other antioxidants such as vitamin E or β-carotene, they can improve lung function in CF patients [208].

## 8. Conclusions

The presence of increased OS and elevated biomarkers of oxidation damage on biomolecules implies that AATD, IPF, and CF patients have a higher demand for antioxidant defence mechanisms. Therefore, targeting OS with antioxidant therapies is a logical approach in these three conditions to delay disease progression and improve patient quality of life. For PCD, the available data are limited, and further studies are required to determine the pathological role of OS in the disease and, therefore, the possibility of antioxidant supplementation. 

As discussed above, several approaches to reduce OS have been explored in animal models or cultured cells in rare respiratory diseases, but few have been tested clinically. Basic research is needed to improve our knowledge of the underlying effects of OS in the lungs to develop more effective antioxidant therapies. Some authors have suggested that antioxidants with relevant therapeutic benefits are molecules that increase the physiological antioxidant response, such as the *Nrf2* activators, and not the antioxidant molecules, which is a promising area for drug development in the future.

As for ER stress-targeting therapy, PERK [209], IRE1 [210], or XBP1 [211,212] knockout mice have lethal consequences. Therefore, blocking primary UPR components is unlikely to be a suitable solution. Nevertheless, some studies suggested the use of drugs that improve the chaperone functions, such as a chemical chaperon 4-phenylbutyrate (4-PBA [213] and tauroursodeoxycholic acid (TUDCA) [214,215], as an alternative to reduce the consequences of ER stress.

## Data Availability

Not applicable.
