# Correction: Magallon et al. Oxidative Stress and Endoplasmic Reticulum Stress in Rare Respiratory Diseases. *J. Clin. Med.* 2021, *10*, 1268

**DOI:** 10.3390/jcm11247322

**Published:** 2022-12-09

**Authors:** María Magallón, Sara Pastor, Ana Esther Carrión, Lucía Bañuls, Daniel Pellicer, Silvia Castillo, Sergio Bondía, María Mercedes Navarro-García, Cruz González, Francisco Dasí

**Affiliations:** 1 Research Group on Rare Respiratory Diseases (ERR), Department of Physiology, School of Medicine, University of Valencia, Avda. Blasco Ibáñez, 15, 46010 Valencia, Spain; 2 Research Group on Rare Respiratory Diseases (ERR), Instituto de Investigación Sanitaria INCLIVA, Fundación Investigación Hospital Clínico Valencia, Avda. Menéndez y Pelayo, 4, 46010 Valencia, Spain; 3 Paediatrics Unit, Hospital Clínico Universitario de Valencia, Avda. Blasco Ibáñez, 17, 46010 Valencia, Spain; 4 Pneumology Unit, Hospital Clínico Universitario de Valencia, Avda. Blasco Ibáñez, 17, 46010 Valencia, Spain

Sara Pastor was not included as an author in the original publication [1]. The corrected Author Contributions Statement appears below. The authors apologize for any inconvenience caused and state that the scientific conclusions are unaffected. This correction was approved by the Academic Editor. The original publication has also been updated.

Dr. Sara Pastor wrote the initial part of the manuscript about 5 years ago.

The manuscript was left unfinished because research work in the laboratory delayed the writing and subsequent publication. Because of the pandemic, access to the lab has been reduced, and we have had more time to devote to article writing; hence, I and the current members of the group have just finished writing the article.

Unfortunately, because Dr. Pastor left the laboratory 4 years ago, I forgot to include her as an author in the manuscript in which she should obviously be listed as an author because she has contributed significantly to the writing of the article.

Dr. Sara Pastor is a co-author of the paper together with Ms. María Magallón.
